# Transfusion-associated adverse reactions (TAARs) and cytokine accumulations in the stored blood components: the impact of prestorage versus poststorage leukoreduction

**DOI:** 10.18632/oncotarget.23136

**Published:** 2017-12-07

**Authors:** Chih-Chun Chang, Tai-Chen Lee, Ming-Jang Su, Hsiu-Chen Lin, Fang-Yi Cheng, Yi-Ting Chen, Tzung-Hai Yen, Fang-Yeh Chu

**Affiliations:** ^1^ Department of Clinical Pathology, Far Eastern Memorial Hospital, New Taipei, Taiwan; ^2^ Department of Nephrology and Division of Clinical Toxicology and Toxicology Laboratory, Chang Gung Memorial Hospital, Lin-Kou Medical Center, Taoyuan, Taiwan; ^3^ College of Medicine, Chang Gung University, Taoyuan, Taiwan; ^4^ School of Medical Laboratory Science and Biotechnology, Taipei Medical University, Taipei, Taiwan; ^5^ Graduate School of Biotechnology and Bioengineering, Yuan Ze University, Taoyuan, Taiwan; ^6^ Department of Medical Laboratory Science and Biotechnology, Yuanpei University, Hsinchu, Taiwan

**Keywords:** cytokines, prestorage leukoreduction, poststorage leukoreduction, febrile nonhemolytic transfusion reactions, transfusion-associated adverse reactions

## Abstract

Leukoreduction in blood units could prevent patients undergoing transfusions from transfusion-associated adverse reactions (TAARs) such as febrile nonhemolytic transfusion reactions (FNHTRs). However, the effect of prestorage and poststorage leukoreduction on TAARs and its underlying mechanisms in stored blood components remains to be determined. Therefore, we investigated the impact of prestorage leukocyte-reduced (pre-LR) and poststorage leukocyte-reduced (post-LR) blood products, including red blood cells (RBCs) and apheresis platelets (PHs), on the incidence of FNHTRs and other TAARs in patients who received transfusions from 2009 to 2014 in a tertiary care center. We also investigated the difference of leukocyte-related bioactive mediators between pre- and post-LR blood components. The results indicated that prevalence of TAARs was significantly reduced in the transfusions of pre-LR blood components. Particularly, the prevalence of FNHTRs was significantly reduced in the pre-LR RBC transfusions and the prevalence of allergy reactions was markedly reduced in the pre-LR PH transfusions. Furthermore, *in vitro* evaluation of cytokines in the pre- and post-LR blood components revealed that IL-1β, IL-8 and RANTES levels were significantly elevated in the post-LR RBCs during the storage. In contrast, IL-1β, IL-6 and IL-8 levels were significantly elevated in the post-LR PHs during the storage. These findings suggested that prestorage leukoreduction had a diminishing effect on the development of TAARs, which could be associated with less accumulation of cytokines in the stored blood components.

## INTRODUCTION

With the advance in transfusion medicine, blood transfusions are considered to be safe in general. However, the risk of complications takes place sometimes during or after transfusion. Of these, febrile nonhemolytic transfusion reactions (FNHTRs) are common transfusion-associated adverse reactions (TAARs), featured with fever (elevated body temperature of 1°C and above 38°C) or chills and rigors, and are usually self-limited in clinical [1]. Besides, it has been reported that FNHTRs occur at a frequency of 0.5–6.8% in all transfused blood units [1–5]. It is believed that leukocytes and the bioactive mediators (mainly cytokines) generated by leukocytes in the transfused blood units are greatly involved in the etiology of FNHTRs and other TAARs [6]. Previous studies revealed that perioperative transfusions of blood components with poststorage leukoreduction prevented complications for those received surgical interventions [7, 8]. Since the 1990s, universal prestorage leukoreduction in blood components has been performed in some advanced countries [9]; and accumulating evidence also indicated that transfusions with prestorage leukoreduced blood units remarkably reduced the rate of FNHTRs [4, 5]. Nevertheless, rare evidence indicated that whether there were significant differences between the effects of prestorage leukocyte-reduced (pre-LR) and poststorage leukocyte-reduced (post-LR) blood components on the frequency of FNHTRs and other TAARs. The expression of cytokines in blood components with prestorage and poststorage leukoreduction, as well as its association with the incidence of TAARs, also remained to be disclosed.

In this study, we retrospectively investigated the impact of prestorage and poststorage leukoreduction of blood components, including red blood cells (RBCs) and apheresis platelets (PHs), on the incidence of FNHTRs and other TAARs in patients who received blood component therapy during the study interval. To investigate the difference of leukocyte-related bioactive mediators between prestorage and poststorage leukoreduction of blood components and find out the underlying mechanisms, we also measured the expression of a variety of cytokines in the blood units with prestorage and poststorage leukoreduction *in vitro*, respectively.

## RESULTS

During the study interval, a total of 4,707 patients underwent allogeneic blood transfusions with 34,301 units. Of these, 1,128 patients were transfused with 10,409 units of pre-LR RBCs, 1,392 were transfused with 13,165 units of post-LR RBCs, 1,490 were transfused with 6,645 units of pre-LR PHs and 697 were transfused with 4,082 units of post-LR PHs. The demographic data were listed in Table 1. In the pre-LR RBCs, 5,970 units were transfused to male and 4,439 to female; and in the post-LR RBCs, 7,039 units were transfused to male and 6,126 to female. The median age of patients receiving pre-LR RBC transfusions was 60 (IQR: 50–72) years, whereas the median age of those undergoing post-LR RBC transfusions was 62 (IQR: 52–75) years. The median day from donor collection to patient transfusion was 12 (IQR: 8–16) days in the pre-LR RBC products, which was less than that in the post-LR RBC products (median days: 19, IQR: 14–24) with statistical significance (*p* < 0.001). On the contrary, 4,072 units were transfused to male and 2,573 to female in the pre-LR PHs, whereas 2,414 units were transfused to male and 1,668 to female in the post-LR PHs. Both the median age of patients receiving pre-LR and post-LR PH transfusions were 57 years; and the median days from donor collection to patient transfusion were 4 days in both pre-LR and post-LR PH transfusions. Furthermore, TAARs were reported in 80 units (0.77%) of the pre-LR RBCs, which were significantly less than those reported to be 135 units (1.03%) in the post-LR RBC transfusions (*p* = 0.039, Table 1). Among these, the unit number with reported FNHTRs was significantly higher in the post-LR RBCs than that in the pre-LR RBCs [65 (0.49%) vs. 24 (0.23%) units, *p* = 0.001]; and between the pre- and post-LR RBC transfusions, there was no significant difference of the unit number with reported allergic reactions [38 (0.37%) vs. 50 (0.38%) units, *p* = 0.850] and other TAARs rather than FNHTRs and allergic reactions [18 (0.17%) vs. 20 (0.15%) units, *p* = 0.680]. Similarly, TAARs were reported in 43 units (0.65%) of the pre-LR PH transfusions, which were also significantly less than those reported to be 47 units (1.15%) in the post-LR PH transfusions (*p* = 0.005). Although there was no statistical significance, the unit number with reported FNHTR was seemingly higher in the post-LR PHs than that in the pre-LR PHs [14 units (0.34%) vs. 12 units (0.18%), *p* = 0.096]. Additionally, the unit number of allergic reactions was significantly higher in the post-LR PHs than that in the pre-LR PHs [30 (0.73%) vs. 26 [0.39%] units, *p* = 0.016]. There was no difference of unit number reported in other TAARs rather than FNHTRs and allergic reactions between the pre- and post-LR PH transfusions [5 (0.08%) vs. 3 (0.07%) units, *p* = 0.970].

**Table 1:** The demographic features of patients undergoing transfusions and the prevalence rate of transfusion-associated adverse reactions

Variables	Leukocyte-reduced RBCs			Leukocyte-reduced PHs		
	Prestorage	Poststorage	*p* value	Prestorage	Poststorage	*p* value
Transfused units	10409	13165		6645	4082	
Male : female	5970:4439	7039:6126	<0.001	4072:2573	2414:1668	0.028
Median age (year)	60 (50–72)	62 (52–75)	<0.001	57 (47–68)	57 (49–66)	0.601
Median days from collection to transfusion	12 (8–16)	19 (14–24)	<0.001	4 (3–4)	4 (4–4)	<0.001
Units with reported transfusion reaction	80 (0.77%)	135 (1.03%)	0.039	43 (0.65%)	47 (1.15%)	0.005
Units with reported of FNHTRs	24 (0.23%)	65 (0.49%)	0.001	12 (0.18%)	14 (0.34%)	0.096
Units with reported of allergic reactions	38 (0.37%)	50 (0.38%)	0.850	26 (0.39%)	30 (0.73%)	0.016
Units with reported of others TAARs	18 (0.17%)	20 (0.15%)	0.680	5 (0.08%)	3 (0.07%)	0.970

Data were expressed as median (interquartile range) or number (percentage). RBC, red blood cell; PH, apheresis platelet; FNHTR, febrile nonhemolytic transfusion reaction; TAAR, transfusion-associated adverse reaction.

To figure out whether patients undergoing transfusions of post-LR blood components had a higher risk of developing TAARs than those receiving pre-LR blood products, conditional logistic regression was analyzed to adjust the confound factors regarding transfusions, as shown in Tables 2 and 3. In all reported RBC TAARs, patients who underwent post-LR RBC transfusions seemed to had a higher risk than those receiving pre-LR RBC transfusions, but without statistical significance (OR: 1.32, 95% CI: 0.97–1.80, *p* = 0.074, Table 2); and there was a significant increased risk of developing FNHTRs in those having post-LR RBC transfusions when compared with the other group receiving pre-LR RBC transfusions (OR: 2.26, 95% CI: 1.35–3.76, *p* = 0.002, Table 2). In contrast, those who receive post-LR PHs had a significantly increased risk of developing TAARs (OR: 1.68, 95% CI: 1.06–2.66, *p* = 0.028, Table 3), especially for mild allergy reactions (OR: 2.03, 95% CI: 1.12–3.69, *p* = 0.020, Table 3), in comparison with the group receiving pre-LR PH transfusions. There was insignificant alteration in mild allergy reactions for those receiving pre- or post-LR RBC transfusions (OR: 0.99, 95% CI: 0.62–1.58, *p* = 0.962, Table 2) and FNHTR for those undergoing pre- or post-LR PH transfusions (OR: 1.32, 95% CI: 0.59–2.97, *p* = 0.506, Table 3). Additionally, the female had a markedly higher risk than the male (OR: 1.55, 95% CI: 1.18–2.04, *p* = 0.001); and those who aged more than 40 years old had a remarkably decreased risk of developing mild allergy reactions in comparison with the group younger than 40 years old in RBC (*p* < 0.001, Table 2) and PH transfusions (*p* = 0.003, Table 3), respectively. It also seemed that patients who received PHs with longer days from donation to transfusion were proned to develop FNHTRs, but there was no statistcal significance (OR: 3.39, 95% CI: 0.96–11.98, *p* = 0.059, Table 3).

**Table 2:** Logistic regression analysis of transfusion-associated adverse reactions with adjustment of confounding factors in leukoreduced red blood cells

Variables	Types of TAARs					
	All reported TAARs	*p* value	FNHTRs	*p* value	Allergy reactions	*p* value
Gender						
Male	1 (reference)	0.001	1 (reference)	0.003	1 (reference)	0.797
Female	1.55 (1.18–2.04)		1.90 (1.24–2.91)		1.06 (0.70–1.62)	
Age (years)						
≤40	1 (reference)	0.119	1 (reference)	0.294	1 (reference)	<0.001
41–60	0.66 (0.43–1.01)	0.055	2.54 (0.91–7.15)	0.077	0.29 (0.17–0.51)	<0.001
61–80	0.72 (0.48–1.10)	0.130	2.36 (0.84–6.62)	0.103	0.27 (0.15–0.47)	<0.001
≥81	0.53 (0.30–0.92)	0.023	1.81 (0.57–5.78)	0.317	0.31 (0.15–0.64)	0.002
Leukoreduction						
Prestorage	1 (reference)	0.074	1 (reference)	0.002	1 (reference)	0.962
Poststorage	1.32 (0.97–1.80)		2.26 (1.35–3.76)		0.99 (0.62–1.58)	
Median days from collection to transfusion (days)						
1–14	1 (reference)	0.890	1 (reference)	0.359	1 (reference)	0.260
15–28	0.96 (0.70–1.30)	0.781	0.93 (0.58–1.49)	0.766	1.02 (0.63–1.66)	0.939
29–42	1.07 (0.63–1.82)	0.795	0.46 (0.16–1.34)	0.154	1.78 (0.85–3.73)	0.127

Data were expressed as median (interquartile range). TAAR, transfusion-associated adverse reaction; FNHTR, febrile nonhemolytic transfusion reaction.

**Table 3:** Logistic regression analysis of transfusion-associated adverse reactions with adjustment of confounding factors in leukoreduced apheresis platelets

Variables	Types of TAARs					
	All reported TAARs	*p* value	FNHTRs	*p* value	Allergy reactions	*p* value
Gender						
Male	1 (reference)	0.694	1 (reference)	0.065	1 (reference)	0.085
Female	0.92 (0.60–1.41)		2.08 (0.96–4.55)		0.60 (0.34–1.07)	
Age (years)						
≥40	1 (reference)	0.004	1 (reference)	0.919	1 (reference)	0.003
41–60	0.42 (0.25–0.72)	0.001	1.06 (0.30–3.76)	0.931	0.33 (0.17–0.62)	0.001
61–80	0.47 (0.27–0.83)	0.008	0.97 (0.26–3.67)	0.965	0.35 (0.18–0.70)	0.003
≥81	0.22 (0.07–0.73)	0.013	1.55 (0.31–7.71)	0.593	N/A	N/A
Leukoreduction						
Prestorage	1 (reference)	0.028	1 (reference)	0.506	1 (reference)	0.020
Poststorage	1.68 (1.06–2.66)		1.32 (0.59–2.97)		2.03 (1.12–3.69)	
Median days from collection to transfusion (days)						
2–3	1 (reference)	0.484	1 (reference)	0.059	1 (reference)	0.703
4–5	1.21 (0.71–2.07)		3.39 (0.96–11.98)		0.88 (0.46–1.70)	

Data were expressed as median (interquartile range). TAAR, transfusion-associated adverse reaction; FNHTR, febrile nonhemolytic transfusion reaction; N/A, not applicable.

*In vitro* study of cytokine concentrations in pre- and post-LR blood components were also performed and the results were shown in Tables 4 and 5 as well as Supplementary Figures 1 and 2. In the RBC components (Table 4, Supplementary Figure 1), cytokine levels of IL-1β, IL-8 and RANTES in post-LR units were significantly increased in comparison with those in pre-LR units on both the 4th day (the initial day of storage) and the 42nd day (the end day of storage). It was also revealed that IL-10 [1.33 (0.07–2.00) vs. 0.00 (0.00–0.30) pg/mL, *p* = 0.002] and MIP-1α [0.43 (0.35–0.67) vs. 0.07 (0.00–0.35) pg/mL, *p* < 0.001] levels in post-LR units were significantly increased on the 4th day when compared with those in pre-LR units. Whereas VEGF [74.19 (57.00–112.04) vs. 7.74 (2.46–9.55) pg/mL, *p* < 0.001] and TGF-β1 [40.32 (28.88–66.56) vs. 7.30 (5.40–11.84) ng/mL, *p* < 0.001], -β2 [2.07 (1.98–5.33) vs. 1.19 (0.96–2.76) ng/mL, *p* = 0.010] and -β3 [1.99 (1.72–2.08) vs. 0.46 (0.35–0.62) ng/mL, *p* < 0.001] levels in post-LR units were significantly increased on the 42nd day in comparison with those in pre-LR units.

**Table 4:** Cytokine expressions in Pre- and Post-LR RBC units on the 4th and 42nd days

Variables	Pre-LR RBCs (*n* = 15)		Post-LR RBCs (*n* = 15)		*p* value	
	the 4th day	the 42nd day	the 4th day	the 42nd day	Pre-LR vs. Post-LR on the 4th day	Pre-LR vs. Post-LR on the 42nd day
IL-1β (pg/mL)	0.09 (0.06–0.33)	0.06 (0.01–0.30)	0.60 (0.19–0.86)	3.85 (3.32–7.72)	<0.001	<0.001
IL-2 (pg/mL)	0 (0–0)	0 (0–0)	0 (0–0)	0 (0–0.56)	0.803	0.263
IL-4 (pg/mL)	0 (0–0)	0 (0–0.06)	0 (0–0.38)	0 (0–0.40)	0.363	0.478
IL-6 (pg/mL)	0 (0–0.16)	0.53 (0–0.76)	0.31 (0–1.43)	0.05 (0–1.91)	0.056	0.984
IL-8 (pg/mL)	0.55 (0.32–1.34)	0.96 (0.63–1.77)	1.16 (0.93–3.50)	2.53 (2.23–6.12)	0.004	0.001
IL-10 (pg/mL)	0 (0–0.30)	0.69 (0.07–1.57)	1.33 (0.07–2.00)	0.68 (0.07–5.05)	0.002	0.711
IFN-γ (pg/mL)	0 (0–0)	0 (0–0)	0 (0–2.53)	0 (0–3.74)	0.064	0.509
MIP-1α (pg/mL)	0.07 (0–0.35)	0.35 (0.27–0.52)	0.43 (0.35–0.67)	0.52 (0.27–0.67)	<0.001	0.395
RANTES (ng/mL)	0.14 (0.09–0.18)	0.10 (0.07–0.13)	0.38 (0.26–0.56)	0.77 (0.47–1.00)	0.001	<0.001
TNF-α (pg/mL)	0.59 (0–1.39)	0 (0–2.31)	0.59 (0–5.06)	0.25 (0–5.56)	0.478	0.453
VEGF (pg/mL)	2.81 (0.00–3.49)	7.74 (2.46–9.55)	5.89 (1.38–16.99)	74.19 (57.00–112.04)	0.052	<0.001
TGF-β1 (ng/mL)	6.88 (5.55–11.31)	7.30 (5.40–11.84)	4.89 (3.23–12.31)	40.32 (28.88–66.56)	0.230	<0.001
TGF-β2 (ng/mL)	1.22 (0.96–2.98)	1.19 (0.96–2.76)	1.38 (1.02–3.43)	2.07 (1.98–5.33)	0.395	0.010
TGF-β3 (ng/mL)	0.46 (0.39–0.68)	0.46 (0.35–0.62)	0.82 (0.63–0.95)	1.99 (1.72–2.08)	0.423	<0.001

Data were expressed as median (interquartile range). RBC, red blood cell; Pre-LR, prestorage leukoreduction; Post-LR, poststorage leukoreduction; IL, interleukin; IFN, interferon; MIP, macrophage inflammatory protein; RANTES, regulated on activation, normal T cell expressed and secreted; TNF, tumor necrosis factor; VEGF, vascular endothelial growth factor; TGF, transforming growth factor.

**Table 5:** Cytokine expressions in Pre- and Post-LR PH units on the 2nd and 5th days

Variables	Pre-LR PHs (*n* = 10)		Post-LR PHs (*n* = 10)		*p* value	
	the 2nd day	the 5th day	the 2nd day	the 5th day	Pre-LR vs. Post-LR on the 2nd day	Pre-LR vs. Post-LR on the 5th day
IL-1β (pg/mL)	0.64 (0.55–0.80)	0.77 (0.42–0.84)	0.98 (0.65–1.56)	1.94 (1.03–3.92)	0.034	0.004
IL-2 (pg/mL)	0 (0–1.09)	0.40 (0–0.95)	0.13 (0–0.68)	0.63 (0–2.30)	0.912	0.003
IL-4 (pg/mL)	0.57 (0.31–0.89)	0.76 (0.42–0.96)	0.78 (0.55–0.84)	0.97 (0.72–1.37)	0.653	0.142
IL-6 (pg/mL)	3.29 (1.77–3.73)	3.87 (2.35–4.57)	4.71 (3.35–5.35)	8.45 (6.12–10.22)	0.031	0.002
IL-8 (pg/mL)	4.62 (4.05–6.01)	5.94 (4.80–6.61)	11.14 (8.35–21.13)	29.15 (12.95–55.17)	0.003	<0.001
IL-10 (pg/mL)	4.51 (2.23–5.62)	3.74 (2.73–4.57)	4.04 (3.08–4.30)	5.61 (3.22–6.72)	0.624	0.271
IFN-γ (pg/mL)	87.72 (57.32–109.02)	90.90 (64.05–105.90)	92.12 (70.68–98.84)	114.75 (91.72–147.10)	0.849	0.121
MIP-1α (pg/mL)	1.36 (1.10–1.42)	1.37 (1.26–1.48)	1.40 (1.18–1.94)	1.94 (1.28–2.47)	0.472	0.075
RANTES (ng/mL)	0.81 (0.63–0.92)	1.37 (0.94–1.43)	0.76 (0.63–0.80)	1.00 (0.94–1.20)	0.569	0.384
TNF-α (pg/mL)	11.38 (6.39–13.23)	11.07 (6.71–13.23)	15.08 (12.00–16.61)	19.21 (15.69–26.05)	0.064	0.004
VEGF (pg/mL)	7.73 (2.88–12.38)	24.51 (13.31–39.91)	1.69 (0.00–5.99)	14.61 (1.04–24.47)	0.046	0.034
TGF-β1 (ng/mL)	19.93 (16.55–43.35)	30.08 (21.96–41.23)	23.03 (19.57–28.47)	30.37 (27.07–37.38)	0.795	0.674
TGF-β2 (ng/mL)	1.63 (1.34–1.74)	1.52 (1.21–1.81)	1.56 (1.43–1.62)	1.61 (1.57–1.76)	0.818	0.211
TGF-β3 (ng/mL)	1.49 (1.47–1.60)	1.99 (1.86–2.03)	1.45 (1.34–1.56)	1.88 (1.76–1.92)	0.327	0.455

Data were expressed as median (interquartile range). PH, apheresis platelet; Pre-LR, prestorage leukoreduction; Post-LR, poststorage leukoreduction; IL, interleukin; IFN, interferon; MIP, macrophage inflammatory protein; RANTES, regulated on activation, normal T cell expressed and secreted; TNF, tumor necrosis factor; VEGF, vascular endothelial growth factor; TGF, transforming growth factor.

In the PH components (Table 5, Supplementary Figure 2), cytokine levels of IL-1β, IL-6 and IL-8 in post-LR units were significantly increased in comparison with those in pre-LR units on both the 2nd day (the initial day of storage) and the 5th day (the end day of storage). Besides, the VEGF level in post-LR units was significantly decreased in comparison with that in pre-LR units on both the 2nd day and the 5th day. The data also revealed that IL-2 [0.63 (0.00–2.30) vs. 0.40 (0.00–0.95) pg/mL, *p* = 0.003] and TNF-α [19.21 (15.69–26.05) vs. 11.07 (6.71–13.23) pg/mL, *p* = 0.004] levels in post-LR units were significantly increased on the 5th day when compared with those in pre-LR units. There was no significant change of other cytokine levels between the pre- and post-LR groups.

## DISCUSSION

Our main findings indicated that the prevalence of TAARs was significantly higher in the transfusion of post-LR blood components then that in the pre-LR blood transfusions. Particularly, the prevalence of FNHTRs was significantly increased in the post-LR RBC transfusions when compared with that in the transfusion of pre-LR RBCs; whereas the prevalence of allergy reactions was remarkably increased in the post-LR PH transfusions when compared with that in the pre-LR PH transfusions. Besides, *in vitro* evaluation of cytokines in the pre- and post-LR blood components revealed that IL-1β, IL-8 and RANTES levels were remarkably increased in the post-LR RBCs when compared with those in the pre-LR RBCs, whereas IL-1β, IL-6 and IL-8 levels were significantly increased in the post-LR PHs when compared with those in the pre-LR PHs during the storage of blood units. It was also shown that VEGF level was significantly decreased in the post-LR PHs when compared with that in the pre-LR PHs. These findings suggested that cytokine accumulations in the blood components were contributed to poststorage leukoreduction and storage of blood units, which could be associated with higher rate of TAARs in patients receiving poststorage leukocyte-depleted blood components when compared with those who were transfused with prestorage leukoreduced ones.

Blood component therapy plays an important role as the life-saving management in certain diseases. Briefly, there were specific storage conditions and preservation solution to extend the shelf-life in each kind of blood components. However, some physicochemical changes take place during the storage of blood components, subsequently affect the overall quality and leading to the TAARs in clinical. During the blood unit storage, generation of proinflammatory cytokines has been believed as one of the manifest causes of TAARs as the clinical consequences [10]. Other lesions derived from the storage of blood components, including increased cell debris and oxidative stress, abnormal rearrangement or loss of cellular membrane phospholipids, as well as morphological alteration of blood cells, could also contribute to the development of TAARs [10–12]. Therefore, leukoreduction was considered to eliminate the adverse effect of blood component preservation. Accumulating evidence has shown that leukoreduction of blood components plays an essential role in decreasing the rate of TAARs and postoperative infection in the transfusion [4, 13–18]. Though it remains controversial [19], universal leukocyte reduction in blood components has been implemented as part of blood safety policy in some economically developed countries [20]. It was observed that transfusion of RBCs and platelet concentrates (PCs) with prestorage leukoreduction greatly reduced the FNHTR rate instead of the rate of allergy reactions [4, 13, 14], partially consistent with our results. However, our results showed that transfusions of pre-LR PHs markedly decreased the rate of allergy reactions and seemingly reduced the events of FNHTR but without statistical significance. These data were consistent with the previous study which demonstrated the rate of allergy reactions was much higher in PH transfusions than that in PC transfusions, while the rate of FNHTRs remained unchanged [21].

Although plenty of investigations indicated the alleviative effect of leukocyte removal in blood components on the rate of TAARs, few studies further disclosed the physicochemical changes of cytokines during the storage and elucidate the role of these proinflammatory markers in triggering TAARs with different blood components. Previously, it was observed the diminishing impact of prestorage leukoreduction on the amount of IL-1, IL-6, IL-8, and TNF-α in the stored RBCs [22]. Further study indicated that the plasma concentration of IL-6 and IL-8, but not IL-1β and TNF-α, was significantly elevated in patients developing FNHTRs during or after RBC transfusions in comparison with the control group [23] While in stored RBCs without leukoreduction, it was noticed that IL-1β, IL-8 and TNF-α levels significantly increased in a time-dependent course [24]. Besides, there was no obvious change in IL-6 concentration during the storage process [24]. Additionally, it was revealed that accumulative VEGF concentration was greatly reduced in the RBCs with prestorage leukoreduction when compared with that in nonleukoreduced RBCs [25]. It was also indicated that prestorage leukoreduction significantly reduced IL-8 concentration on storage of canine RBC concentrates [26]. Moreover, it was reported that IL-8, RANTES and epidermal growth factor (EGF) levels as well as the expressions of soluble CD40 ligand (sCD40L) and soluble Fas ligand (sFas-L), two members of the TNF family, were significantly reduced in the whole blood with prestorage leukoreduction [27]. However, most cytokine levels such as granulocyte-macrophage colony-stimulating factor (GM-CSF), IFN-γ, IL-2, IL-5, IL-10, IL-13, IL-15 and IL-17, remained unaltered or decreased during the storage period in the whole blood without leukoreduction [28]. These findings in RBC products were generally consistent with our results that cytokine levels of IL-1β, IL-8 and RANTES were significantly decreased by prestorage leukoreduction during the storage. In contrast, remarkable elevation of IL-1β, IL-6, IL-8 and TGF-β1 levels was seen during the process of storage in PCs [28, 29]. Furthermore, it was reported that several cytokines, including RANTES, TGF-β1, IL-27, as well as soluble OX40 ligand (sOX40L), also a member of the TNF family and being associated with the inhibition of IL-6, IL-10 and IL-12 [30], were significantly increased during the storage of PHs [31–33]. These findings in the platelet products were partially compatible with our results since our data indicated that cytokine levels of IL-1β, IL-6 and IL-8 levels were significantly reduced by prestorage leukoreduction during the storage. Although the cytokine levels of RANTES and TGF-β1 were seemingly decreased in prestorage leukoreduction, there was no statistical significance in comparison with those in poststorage leukoreduction.

Interestingly, our current results showed the impact of prestorage leukoreduction on reducing the accumulative level of VEGF in both RBCs and PHs, which was compatible with the previous investigation [25]. However, the VEGF level was somewhat lower in the post-LR PHs than that in the pre-LR PHs at both the initial and the end days of storage. It was known that serum VEGF was mainly released from platelets [34], but the underlying mechanism of reduced VEGF concentration in the post-LR PHs remains unclear. This phenomenon may be partially explained by the different storage termperature and environment of RBC and PH components. Generally, RBC products were stored at 1–6°C, while platelet products were stored at room temperature, which may result in VEGF degeneration in a time-depedent manner. It was also reported that cytokine accumulations may be inversely correlated with the expression of VEGF as certain cytokines were not downregulated till the response to VEGF was presented, which was partially consistent with our data [35]. Further research is required for demonstration and integration of the storage effect and the difference of prestorage and poststorage leukoreduction on the cytokine cascades in various blood components. Moreover, though we noticed that certain cytokines accumulated in a time-dependent manner during the storage, we did not observe a higher rate of TAARs in patients who received stored blood components with longer days of donation to transfusion. One possible explanation is the inadequate awareness and under-reporting rate for the surveillance of TAARs, resulting in selection bias to some extent. Nevertheless, it was sometimes difficult to differentiate the TAARs from preexisted comorbidities and disease progression in certain groups. These may imply why it revealed no obivous correlations between the incidence of TAARs and the interval from donation to transfusion in the stored blood components in patients receiving transfusion therapy in our data.

There were several limitations in our investigation. One of the major methodological limitations was that the prospective data collection on clinical TAARs was retrospectively analyzed in our study. Besides, the blood unit number for *in vitro* evaluation of cytokine expressions in RBCs and PHs was not large enough, and could thus lead to selection bias to a certain extent. Besides, cytokine expressions were not surveyed in patients who had TAARs before and after trnasfusions, making it difficult to link TAARs with higher level of certain cytokines in direct. Moreover, our study merely focused on the effect of prestorage and poststorage leukoreduction on the alteration of the proinflammatory factors and the evaluation of their accumulative changes in various stored blood units. Hence, the association of blood cell-derived extracellular bioactive microvesicles and phospholipids of the cellular membrane in blood units with storage and leukoreduction was not further evaluated. It was reported that these extracellular microvesicles from both fresh and stored RBCs could be implicated with inflammatory host response, leading to production of TNF, IL-6 and IL-8 [36]. Additionally, human platelet antigens (HPAs), which were reported to be associated with FNHTR evocation in patients suffering from infectious diseases [37], were also not surveyed in this study. Further research is warranted to explore the exact mechanisms in each kind of blood component therapy-induced adverse reactions.

In summary, our results indicated that prevalence of TAARs was significantly reduced in the transfusions of pre-LR blood units when compared with that in the transfusions of post-LR blood components. Particularly, the prevalence of FNHTRs was significantly reduced in the pre-LR RBC transfusions when compared with that in the transfusion of post-LR RBCs and the prevalence of allergy reactions was markedly reduced in the pre-LR PH transfusions when compared with that in the pre-LR PH transfusions. Furthermore, *in vitro* evaluation of cytokines in the pre- and post-LR blood components revealed that IL-1β, IL-8 and RANTES levels were significantly elevated in the post-LR RBCs when compared with those in the pre-LR RBCs during the storage. In contrast, IL-1β, IL-6 and IL-8 levels were significantly elevated in the post-LR PHs when compared with those in the pre-LR PHs during the storage. These findings suggested that prestorage leukoreduction had a diminishing effect on the development of TAARs, which could be associated with less accumulation of cytokines in the stored blood components.

## MATERIALS AND METHODS

From July, 2009 to December, 2014, adult patients (aged more than 20 years old) who had received blood components with leukoreduction in Far Eastern Memorial Hospital (FEMH) were registered. Transfusion of leukocyte-depleted blood components was indicated for patients with organ or bone marrow transplantation, immunocompromised status, or who had experienced FNHTR in previous transfusions. The clinical data, such as patient age, gender, transfusion of blood components (including RBCs or PHs; pre- or post-LR blood products), storage days from collection to transfusion and experience of TAARs, were requested via the chart review of electronic medical record for further analysis. The pre-LR blood components were mainly supplied by Taiwan Blood Services Foundation (TBSF). Whereas the post-LR blood products were collected with the performance of leukocyte filtration on the non-LR components supplied by TBSF. All post-LR procedures were executed with in-line leukocyte filters (BioR-plus and BioP-plus, Fresenius Kabi AG, Homburg, Germany) [38] to achieve a leukocyte reduction of less than 5 × 10^6^ residual leukocytes per unit in the Blood Bank, Department of Clinical Pathology, FEMH, according to the American Association of Blood Banks (AABB) criteria [39]. The storage days of pre- and post-LR blood products from blood donor collection to bedside transfusion were also recorded. The pre- and post-LR blood products were randomly selected and assigned for transfusion at request. This study was approved by the research ethics review committee of FEMH.

TAARs were considered and reported to the blood bank in FEMH via a hemovigillance system, supposing that patient had experienced one or more following symptoms during or after cessation of blood transfusion: fever, chills/rigors, urticaria, skin itching, dyspnea/tachypnea, headache, nausea/vomiting, hypotension/hypertension, backache, hematuria, oligouria, and so on. The reported TAARs were surveyed and ascertained by at least two blood bank physicians, according to the National Healthcare Safety Network (NHSN) Biovigilance Component Hemovigilance Module Surveillance Protocol [40]. Briefly, development of pruritic urticaria during or within 4-hour of transfusion cessation was considered as a minor allergic reaction. Besides, a FNHTR was considered if the body temperature was noticed to be 38°C and more, accompanied with an elevation of at least 1°C when compared with baseline value, or development of chills/rigors was observed during or within 4 hours of transfusion cessation. Besides, other TAARs were categorized into others rather than FNHTRs and allergic reactions.

To determine the cytokine levels in blood components with prestorage and poststorage leukoreduction, plasma were separated from a sum of 2 mL extracted blood sample from each blood unit, with centrifugation at 1000 × g for 15 minutes at 4°C, and were then collected and stored at −70°C. A total of 15 units of pre-LR and 15 units of post-LR RBCs, all were blood type O and Rh positive and were suspended in citrate-phosphate-dextrose-saline-adenine-glucose-mannitol (CPD-SAGM) solution at 4°C, as well as 10 units of pre-LR and 10 units of post-LR PHs, all were blood type O and Rh positive and were suspended in anticoagulant-citrate-dextrose solution A (ACD-A) at the room temperature with continuous gentle agitation, were used for the measurement of cytokines. In both pre-LR and post-LR RBCs, blood samples were extracted on the initial day (the 4th day) and the end day (the 42nd day) of storage for each unit, respectively. Whereas in both pre-LR and post-LR PHs, blood samples were extracted on the initial day (the 2nd day) and the end day (the 5th day) of storage for each unit, respectively.

The magnetic beads-based Bio-Plex Pro^TM^ human cytokine 17-plex assay was carried out on the Luminex 200 System (Bio-Rad Laboratories, Philadelphia, US), according to the manufacturer's maneuvers. Briefly, a variety of cytokines was measured, including interleukin (IL)-1β, IL-2, IL-4, IL-6, IL-8, IL-10, interferon (IFN)-γ, macrophage inflammatory protein (MIP)-1α, regulated on activation, normal T cell expressed and secreted (RANTES), tumor necrosis factor (TNF)-α, vascular endothelial growth factor (VEGF) and transforming growth factor (TGF)-β1, -β2 and -β3. Before the evaluation of cytokines, plasma samples were diluted as 1:3 in the Bio-Plex sample diluents. The reconstitution and serial dilutions of standard were performed for subsequent evaluation. The median fluorescence intensity (MFI) of each specimen was detected and recalculated into the cytokine concentrations with the optimized standard curve using the Bio-Plex software (version 6.0, Bio-Plex Manager). The detected values of MFI less than the lower limit of detection would be considered as undetectable and thus be described as zero for statistical analysis.

Statistical analysis was performed by SPSS (version 19.0; SPSS Inc., Chicago, USA) statistical software. Demographic data were presented by the median (interquartile range, IQR) using descriptive statistics. The comparison of the median days from collection to transfusion of blood products as well as the expression of cytokines in each group was analyzed by Mann-Whitney *U* test. The prevalence of transfusion-associated adverse reactions in each group was analyzed and compared by Pearson's chi-squared test or Fisher's exact test. Conditional logistic regression analysis was performed in controlling for confounding factors regarding blood component-specific TAARs with the expression of odds ratio (OR) with 95% confidence interval (CI). Statistical significance was considered if the two-tail *p* value was less than 0.05.

## SUPPLEMENTARY FIGURES


