# Supplementary material for: Transfusion-associated adverse reactions (TAARs) and cytokine accumulations in the stored blood components: the impact of prestorage versus poststorage leukoreduction
Source: Oncotarget. 2017 Dec 7;9(4):4385–94. doi: 10.18632/oncotarget.23136 (PMC5796981; doi:10.18632/oncotarget.23136)
Supplement: Supplementary file 1 [file oncotarget-09-4385-s001.pdf]

Transfusion-associated adverse reactions (TAARs) and cytokine accumulations in the stored blood components: the impact of prestorage versus poststorage leukoreduction

SUPPLEMENTARY MATERIALS

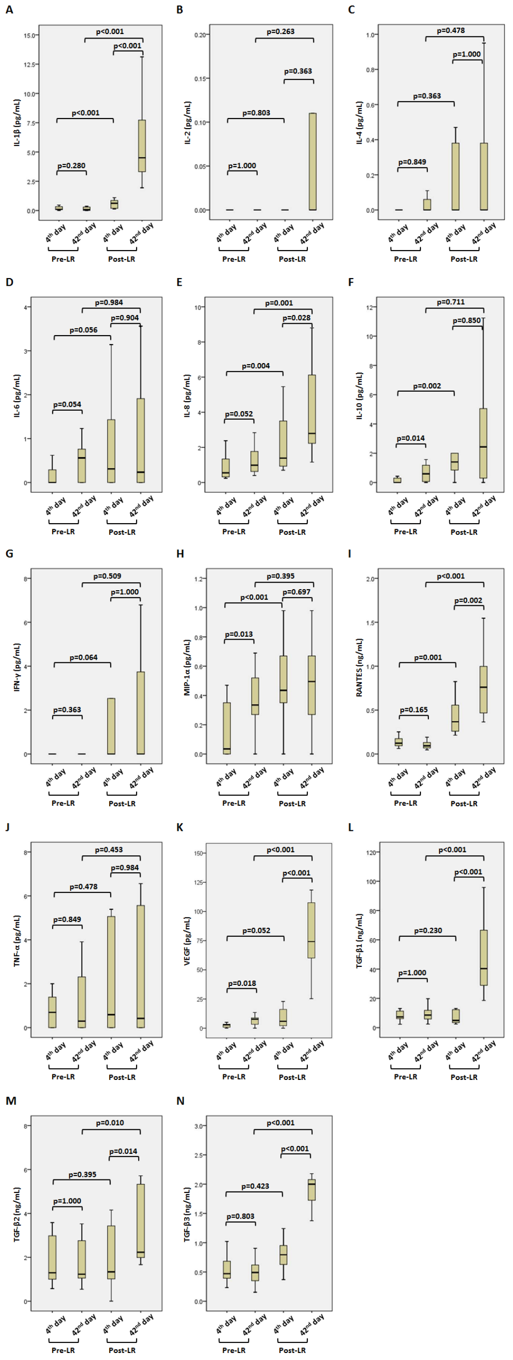

Supplementary Figure 1: *In vitro* study of cytokine expressions in prestorage (pre-) and poststorage (post-) leukocyte-reduced (LR) red blood cells (RBCs) on the initial day (the 4th day) and the end day (the 42nd day) of storage. (**A**) interleukin (IL)-1β; (**B**) IL-2; (**C**) IL-4; (**D**) IL-6; (**E**) IL-8; (**F**) IL-10; (**G**) interferon (IFN)-γ; (**H**) macrophage inflammatory protein (MIP)-1α; (**I**) regulated on activation, normal T cell expressed and secreted (RANTES); (**J**) tumor necrosis factor (TNF)-α; (**K**) vascular endothelial growth factor (VEGF); (**L**) transforming growth factor (TGF)-β1; (**M**) TGF-β2; (**N**) TGF-β3 (n = 15 in each group). A p value less than 0.05 was considered statistically significant.

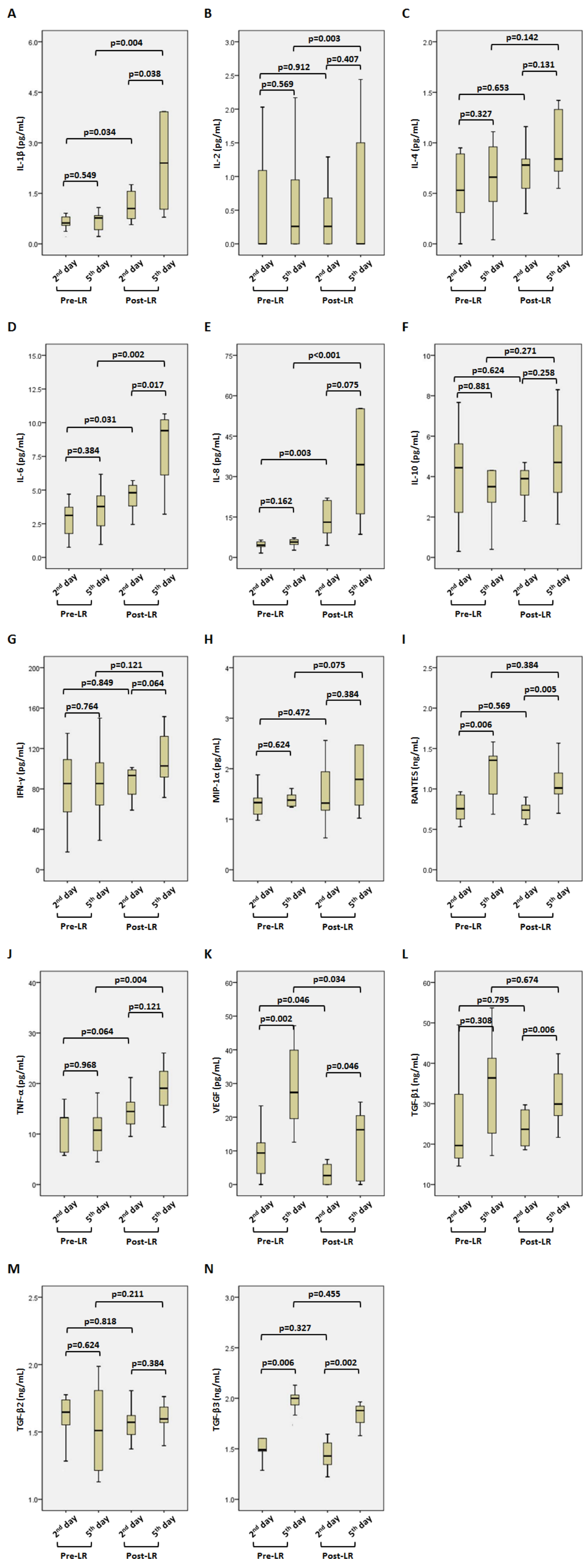

Supplementary Figure 2: *In vitro* study of cytokine concentrations in prestorage (pre-) and poststorage (post-) leukocyte-reduced (LR) apheresis platelets (PHs) on the initial day (the 2nd day) and the end day (the 5th day) of storage. (**A**) interleukin (IL)-1β; (**B**) IL-2; (**C**) IL-4; (**D**) IL-6; (**E**) IL-8; (**F**) IL-10; (**G**) interferon (IFN)-γ; (**H**) macrophage inflammatory protein (MIP)-1α; (**I**) regulated on activation, normal T cell expressed and secreted (RANTES); (**J**) tumor necrosis factor (TNF)-α; (**K**) vascular endothelial growth factor (VEGF); (**L**) transforming growth factor (TGF)-β1; (**M**) TGF-β2; (**N**) TGF-β3 (n = 10 in each group). A p value less than 0.05 was considered statistically significant.
